# Time-series transcriptomic analysis of the kelp grouper *Epinephelus moara* in response to low salinity stress

**DOI:** 10.1080/19768354.2018.1487335

**Published:** 2018-08-22

**Authors:** Quanxin Gao, Yanfeng Yue, Minghua Min, Shiming Peng, Zhaohong Shi, Jinbo Wang, Tao Zhang

**Affiliations:** a Key Laboratory of Marine and Estuarine Fisheries, Ministry of Agriculture, East China Sea Fisheries Research Institute, Chinese Academy of Fishery Sciences, Shanghai, People’s Republic of China; b Ningbo Institute of Technology, Zhejiang University, Ningbo, People’s Republic of China; c Aquatic Technology Promoting Station of Meijiang District, Meizhou, People’s Republic of China

**Keywords:** *Epinephelus moara*, transcriptome, low-salinity stress

## Abstract

The Kelp grouper *Epinephelus moara* is one of the most widely consumed and economically important marine fish in China. The species can tolerate a wide range of salinity, but genomic resources are not available, and the molecular mechanisms underlying adaptation to salinity at the transcriptomic level remain largely unclear. In this study, the transcriptomic responses of the liver of *E. moara* under low salinity were investigated using the Illumina digital gene expression system. After *de novo* assembly, 499,356 transcripts were generated and contributed 445,068 unigenes. A total of 14, 19, 33 and 3101 genes were differentially expressed following exposure to low salinity stress for 2, 6, 24 and 48 h, respectively. Only two genes were differentially expressed in all groups. Four genes related to metabolism and ambient salinity adaption were randomly selected to validate the differentially expressed genes (DEGs) by real-time PCR. Gene Ontology (GO) and Kyoto Encyclopaedia of Genes and Genomes (KEGG) pathway enrichment analysis were used to analyse the functional significance of DEGs, including those responding to salinity through diverse biological processes, cellular components, molecular functions, and pathways associated with metabolic and osmotic responses. This work provides new insight into the response to salinity challenges in *E. moara*, and the findings expand our knowledge of the molecular basis of metabolic regulation mechanisms in this species. Additionally, the transcriptional data provide a valuable resource for future molecular and genetic studies on *E. moara*.

## Introduction

The Kelp grouper *Epinephelus moara* is a large marine fish that inhabits the bottom of warm oceans in the western North Pacific, particularly those surrounding Vietnam, southern and eastern China, South Korea and Japan (Guo et al. 2009). The meat of this species is renowned for its delicious, fresh taste, and it is nutrient-rich, making it a favourite of consumers and bestowing high economic value. Due to the limited abundance of *E. moara* populations and overfishing, the species is gradually becoming scarce and the price keeps rising. In China, many researchers have carried out artificial breeding and seed cultivation experiments on *E. moara*, and found that it grows fast and possesses high adaptability (Tian et al. 2017a). The fish is an important marine aquaculture species in south-eastern coastal regions of China.

Salinity is an environmental factor affecting osmotic pressure regulation in fish that directly influences metabolism and immune responses, restricting the distribution of fish in natural ecosystems. In mariculture, salinity has a significant effect on the development of fish embryos, larvae and juveniles (Sørensen et al. 2016; Kujawa et al. 2017). It affects the ability of grouper to regulate osmotic pressure, the activity of metabolic enzymes, and the abundance of immune-related factors, influencing the ability to regulate osmotic pressure, digestion and absorption of nutrients, resistance to disease, and ultimately growth and survival (Tsui et al. 2012; Cheng et al. 2013; García et al. 2013; Chapman et al. 2014; Sutthinon et al. 2015; Chen et al. 2016; Arrokhman et al. 2017). Therefore, studying the effects of salinity on the growth and development of grouper is important for artificial breeding programmes. Due to the widely fluctuating salinity in coastal areas of China, especially during the breeding season when rainfall can be high and salinity low in estuaries, the growth and survival of aquatic animals can be restricted. Therefore, studying the impact of low salinity stress on *E. moara* is important.

Salinity is an important factor in the successful recruitment of larval and juvenile groupers (Gracia-López et al. 2004; Cheng et al. 2013). Various enzymes, transporters and signal transmission pathways participate in the processes of salinity adaption and osmoregulation to maintain an internal osmotic and ionic homeostasis when live in a salinity changing environment (Tsui et al. 2012; Sutthinon et al. 2015; Chen et al. 2016). It is believed that the drastic change of liver metabolism can be triggered by changing the salinity (Li et al. 2017). Analysis and identification of candidate genes involved in salinity change is the first step to elucidate the molecular mechanism and understand factors underlying this core physiological process. Transcriptomics can be used to investigate the transcriptional regulation of all genes in cells and tissues. The development and application of transcriptomics approaches can not only reveal details of the cellular components, but also help to elucidate the functional genes and components in the genome, which are of great importance for studying the growth and immune responses of organisms (Meng et al. 2015; Cao et al. 2017; Zhang et al. 2017). In recent years, transcriptomics has been applied by aquaculture researchers to study the effects of salinity stress on aquatic animals (Thanh et al. 2014). In the present study, next-generation high-throughput sequencing was used to analyse key genes and signal transduction pathways related to salinity stress in *E. moara*. The findings deepen our understanding and will be of benefit to grouper breeding programmes and controlling diseases during aquaculture of this important food species.

## Materials and methods

### Experimental fish

*E. moara* were obtained from the Yellow Sea Fisheries Research Institute. This study was conducted at the Shanghai Fisheries Research Institute. Fish with a similar body length (10.43 ± 1.08 cm) and weight (16.73 ± 4.47 g) were acclimatised in aerated normal seawater with a salinity of 27% for 2 weeks before reduced salinity exposure, during which 25% of the tank water was renewed daily. After acclimatisation, 60 healthy fish were randomly divided into three tanks and exposed to a low salinity of 15 ppt. Three individual fish from each tank were randomly sampled after exposure to the reduced salinity conditions for 0, 2, 6, 24, and 48 h. Liver samples were collected and immediately flash-frozen in liquid nitrogen and stored at -80°C until they were processed for RNA extraction.

### RNA extraction and library preparation for Illumina sequencing

Total RNA from livers was extracted using Trizol reagent (Invitrogen, USA) according to the manufacturer's instructions. RNA quantity, purity and integrity were determined by 1% agarose gels and a Nanodrop 1000 spectrophotometer (NanoDrop, USA). High-quality RNA was selected for high-throughput sequencing, and samples from the three fish for each time point were combined. mRNA was purified from total RNA using oligo-dT-attached magnetic beads and fragmented using divalent cations under elevated temperature. First-strand cDNA was synthesised using random primers and reverse transcriptase. Second-strand cDNA synthesis was subsequently performed using DNA polymerase I and RNase H. A paired-end library was constructed using the Genomic Sample Prep Kit and sequenced using the Illumina Mi-Seq platform (Yin et al. 2016).

### Assembly and annotation of the transcriptome

Raw sequencing reads with an average length of 250 bp were generated and filtered through the Illumina sequencing platform. All dirty raw reads with adaptors, unknown bases at >10%, and low-quality sequences were trimmed to generate a set of clean reads. Short sequences were also removed using a custom Perl Program. The resulting high-quality sequences stemming from quality control analysis were *de novo* assembled into contigs and transcripts with Trinity software. To reduce data redundancy and evaluate the efficacy of the assembly, transcripts with a minimum length of 200 bp were assembled and clustered using TGICL under default parameters. The longest transcript among the multitude of assembly transcript isomers was defined as a unigene. All unigenes were used as queries for searching nr, nt and Swiss-Prot databases, and functionally annotated by Gene ontology (GO) and Kyoto Encyclopaedia of Genes and Genomes (KEGG) pathway analysis (Song et al. 2016).

### Identification of differentially expressed genes (DEGs)

The number of reads aligned to each unigene in the alignment file and the read counts were normalised as reads per kilobase per million (RPKM) values. The false discovery rate (FRD) was used to determine the threshold of the *p*-value in multiple tests to judge the significance of gene expression differences. DEGs between pairwise comparisons (0 h vs. 2 h, 0 h vs. 6 h, 0 h vs. 24 h, and 0 h vs. 48 h) were identified using the Bioconductor tool edgeR (*p*-value <0.05, FDR <0.05). Functional enrichment analysis of DEGs was performed by mapping each DEG to GO and KEGG databases. The FDR was used to correct the *p*-value and, pathways with FDR values <0.05 were considered significantly enriched (Thanh et al. 2014; Nie et al. 2016).

### Quantitative real-time PCR (qRT-PCR) analysis

To validate our Illumina sequencing data, four genes were randomly selected for *q*RT-PCR analysis using a SYBR Premix Ex Taq kit (Invitrogen) according to the manufacturer's instructions. The same pooled RNA samples used for generating RNA-seq data were reverse-transcribed into cDNA using a PrimeScript 1^st^ strand cDNA synthesis kit (Takara, Japan). Specific primers used for *q*RT-PCR were designed using Primer 5 software and are listed in Table 1. The *β*-actin gene was chosen as an endogenous control. Relative gene expression was determined using the 2^−△△Ct^ method (Gao et al. 2016; Yin et al. 2016).

**Table 1.** Sequences of primers used in real-time PCR assays.

Target Genes	Primer Sequences		Size (bp)
TRAF-type zinc finger domain-containing protein 1-like	F (5′-3′)	GGAGAAGGTTGGGTAGTTG	120
	R (5′-3′)	GCTGGAGGTGTAGAGGGA	
Parvalbumin beta	F (5′-3′)	CTTGTCTGTATCATCTGTACCCTAC	127
	R (5′-3′)	CGAATGTGCCAGCGTCT	
SH3 domain-binding glutamic acid-rich-like protein 3-like	F (5′-3′)	TGCCGCTCACAGTGTTT	159
	R (5′-3′)	CTGCCAACTTCCTCATTACA	
Cold shock domain-containing protein E1	F (5′-3′)	GCATTGGAGGCATTACG	107
	R (5′-3′)	TACCCACAGGTTGGAGACA	
*β*-actin	F (5′-3′)	TGCTGCTTGAGTTTCTACAT	117
	R (5′-3′)	CCCACTGTCTGCGATTA	

## Results

### Transcriptome sequencing and de novo assembly

A total of 63,545,722, 37,741,048, 49,822,946, 64,207,362 and 126,858,410 raw reads were generated from the 0, 2, 6, 24 and 48 h groups, respectively. After removing reads containing adapter, reads containing poly-N sequences, and low-quality reads, 61,757,036, 36,311,164, 47,969,516, 62,429,208 and 124,017,584 clean reads with an average length of 140, 139, 142, 141 and 138 bp were obtained for subsequent analysis. The percentages of aligned reads were 57.3%, 55.9%, 66.9%, 60.4% and 51.7%, respectively. Further assembly analysis showed that these high-quality reads generated 499,356 transcripts and contributed to 445,068 unigenes. The quality of these reads is shown in Tables 2 and 3.

**Table 2.** Statistical analysis of raw and preprocessed sequences.

Samples	Raw reads	Raw bases	Trimmed reads	Trimmed bases	Average length (bp)	Trimmed reads %	Trimmed bases %
0 h	63545722	9531858300	61757036	8633992960	140	97.2	90.6
2 h	37741048	5661157200	36311164	5035138564	139	96.2	88.9
6 h	49822946	7473441900	47969516	6834476368	142	96.3	91.5
24 h	64207362	9631104300	62429208	8813244643	141	97.2	91.5
48 h	126858410	19028761500	124017584	17129141793	138	97.8	90.0

**Table 3.** Summary of *de novo* assembly of transcriptomic profiles.

Samples	Unigenes	Transcripts	Aligned reads	Unaligned reads	Aligned rate (%)	Unaligned rate (%)
0 h	190163	212587	35392662	26364374	57.3	42.7
2 h	149770	168506	20299096	16012068	55.9	44.1
6 h	175551	196690	32111410	15858106	66.9	33.1
24 h	204610	227903	37683502	24745706	60.4	39.6
48 h	327461	369717	64141958	59875626	51.7	48.3

### Identification and analysis of DEGs

**Figure 1.**
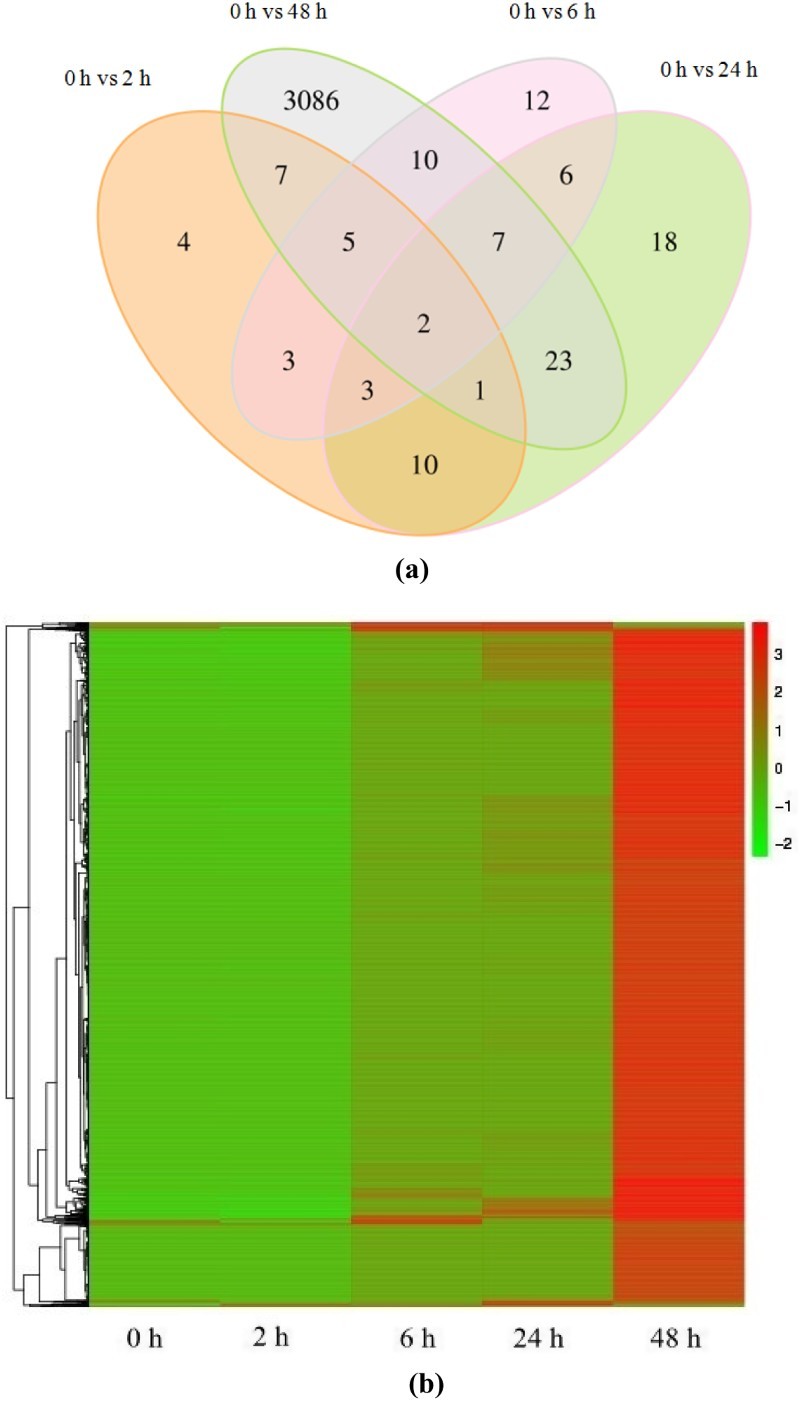
Comparison of DEGs identified by microarray analysis of *Epinephelus moara* under low-salinity stress for different durations. (a) Venn diagram showing the number of DEGs present at all four time points. (b) Heat map summarising DEGs identified at different time points.

Overall, 3294 genes from the four time point groups were significantly differentially expressed in response to salinity acclimation. Of these, 3167 were up-regulated significantly (*p*-value <0.05), comprising 14 (0 h vs. 2 h), 19 (0 h vs. 6 h), 33 (0 h vs. 24 h) and 3101 (0 h vs. 48 h) genes in the four respective groups. The number of genes down-regulated (*p*-value <0.05) was 127, comprising 21, 29, 37 and 40 genes in the 2, 6, 24 and 48 h groups (Table 4). However, only two DEGs were present in all pairwise comparisons (Figure 1(a)). Many more DEGs (3167) were up-regulated between 0 and 48 h (Table 4), and a wide variety of genes were up-regulated following salinity stress (Figure 1(b)).

**Table 4.** Number of DEGs, GO terms and KEGG pathways (*p*-value <0.05) from pairwise comparisons.

Pairwsie comparisons	DEGs up-regulated	DEGs down-regulated	∑	GO terms				KEGG pathways
				Biological process	Cellular component	Molecular function	∑	
0 h vs. 2 h	14	21	35	315	81	58	454	23
0 h vs. 6 h	19	29	48	185	42	38	265	4
0 h vs. 24 h	33	37	70	184	57	67	308	28
0 h vs. 48 h	3101	40	3141	1427	188	540	2155	95
∑	3167	127	3294	2111	368	703	3182	150

### GO analysis

GO annotation, a standardised gene functional classification system, applied to assign annotated genes to biological process, and molecular and cellular component categories. A total of 445,068 unigenes were annotated by GO and classified into 22,285 sub-categories, including 15,158 terms for biological process, 1857 terms for cellular component, and 5270 terms for molecular function. Between 0 and 2 h, significantly enriched DEGs were related to 454 GO terms (*p*-value <0.05), comprising 315 biological process, 81 cellular component, and 58 molecular function categories (Table 4). The cellular component category was highly represented by MHC class II protein, complex MHC protein and endosome sub-categories. The biological process category was highly represented by peptide antigen binding, phosphate ion binding, and antigen binding sub-categories, while antigen processing and presentation of exogenous peptide antigen via MHC class II, peptide antigen via MHC class II, and peptide or polysaccharide antigen via MHC class II were the dominant biological process sub-categories (Figure 2(a)). Figure 2.The most enriched GO terms and KEGG pathways of DEGs identified in the liver of *E. moara* in response to low-salinity stress. The most significantly enriched GO terms of DEGs identified in the comparisons of the 0 and 2 h (a), 0 and 6 h (c), 0 and 24 h (e), 0 and 48 h (g) time points are displayed using histograms. The vertical axis represents the top 10 most significant GO terms in the three main GO categories (biological process, cellular component, and molecular function). The horizontal axis indicates the -log2(*p*-value) of GO terms. The top 30 most significant KEGG pathways identified in the comparison of the 0 and 2 h (b), 0 and 6 h (d), 0 and 24 h (f), 0 and 48 h (h) time points are also presented using histograms. The vertical axis represents the pathway category, and the horizontal axis indicates the -log2(*p*-value) of significant pathways.

**Figure:**
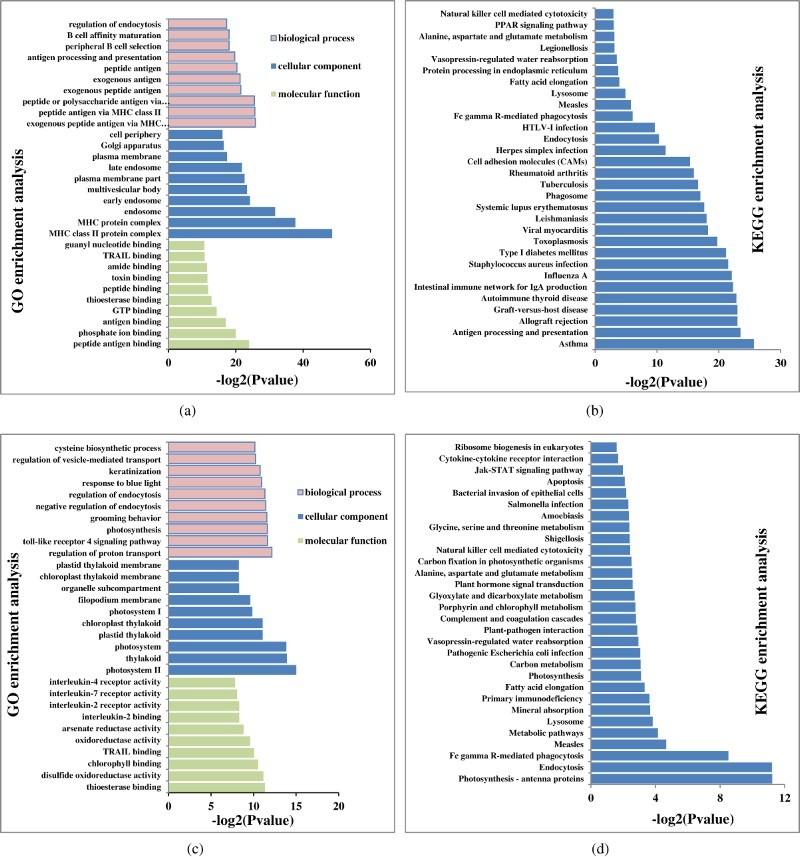

**Figure:**
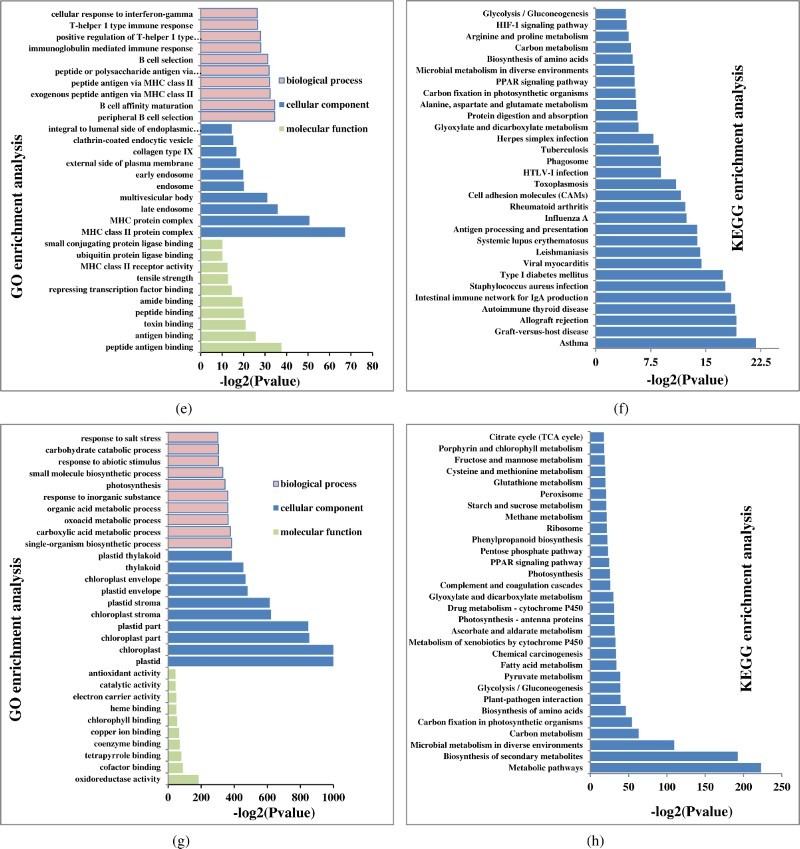

In the 0 and 6 h pairwise comparison, 265 GO terms were significant (*p*-value <0.05), including 38 molecular function, 42 cellular component, and 185 biological process sub-categories (Table 4). In the 0 and 24 h pairwise comparison, 308 GO terms were identified (*p*-value <0.05), including 184 biological process, 57 cellular component, and 67 molecular function sub-categories (Table 4). Furthermore, in the 0 and 48 h pairwise comparison, the number of GO terms (*p*-value <0.05) was increased significantly (2155), including 1427 biological process, 188 cellular component, and 540 molecular function sub-categories (Table 4). In general, GO terms identified in the 0 and 48 h pairwise comparison were much more significant than from other pairwise comparisons. The top 10 most enriched GO terms in the three main GO categories (biological process, cellular component and molecular function) among all pairwise comparisons are shown in Figure 2Figure 2(a, c, e and g).

### KEGG analysis

KEGG pathway analysis was performed to investigate pathways that were significantly altered following exposure to low salinity. The most enriched KEGG pathways are shown in Figure 2(b, d, f and h). There were 115 significant KEGG pathways identified, including 23 in the 0 and 2 h pairwise comparison, 4 in the 0 and 6 h pairwise comparison, 28 in the 0 and 24 h pairwise comparison, and 95 in the 0 and 48 h pairwise comparison (Table 4). Metabolic pathways (-log2[*p*-value] = 223) was the most significant KEGG pathway in the 0 and 48 h pairwise comparison, followed by biosynthesis of secondary metabolites (-log2[*p*-value] = 192) and microbial metabolism in diverse environments (-log2[*p*-value] = 110). These three KEGG pathways all belong to the metabolism pathway class, indicating that metabolism in *E. moara* was significantly affected after a 48 h exposure to low salinity conditions.

### Validation of RNA-seq data by real-time PCR

**Figure 3.**
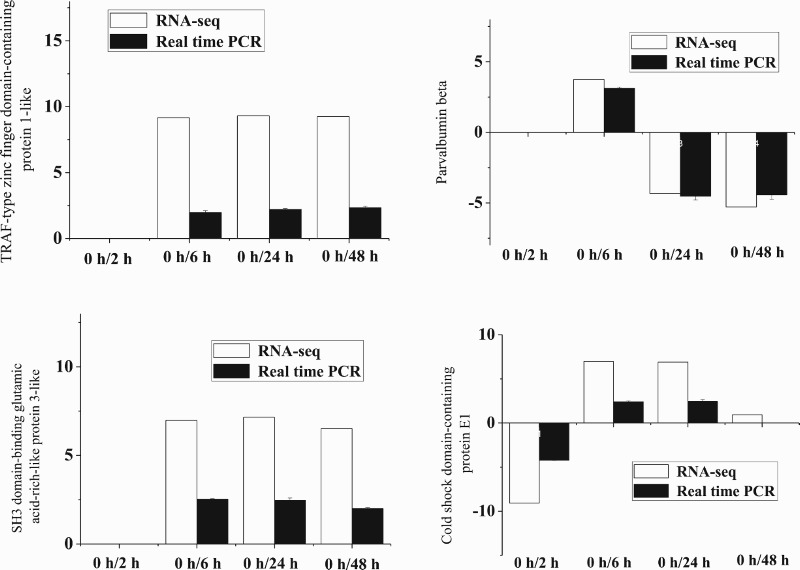
Validation of RNA-seq data by real-time PCR. The expression of TRAF-type zinc finger domain-containing protein 1-like, parvalbumin beta, SH3 domain-binding glutamic acid-rich-like protein 3-like, and cold shock domain-containing protein E1 was measured by RNA-seq (white columns) and real-time PCR (black columns) and plotted as -log2(fold change).

To validate the sequencing data, four unigenes, namely TRAF-type zinc finger domain-containing protein 1-like, parvalbumin beta, SH3 domain-binding glutamic acid-rich-like protein 3-like, and cold shock domain-containing protein E1, were randomly selected for real-time PCR experiments using the same pooled RNA samples employed for generating the RNA-seq data. The expression levels of these four genes measured by RNA-seq and real-time PCR are presented in Figure 3. The expression levels of all four genes measured by real-time PCR were consistent with those of RNA-seq, confirming the validity of the RNA-seq results.

## Discussion

Salinity is an important factor in the marine environment that can have a direct impact on the growth and development, reproduction, and immunity of fish and other marine organisms. Changes in salinity can force fish to adjust the dynamic balance of the osmotic pressure through a series of physiological changes, which will lead to corresponding changes physiological and biochemical processes such as digestion and absorption of food, nutrient metabolism, and immunoregulation (Tran-Ngoc et al. 2017; Tian et al. 2017b). Salinity changes can directly affect the activity of enzymes regulating osmotic pressure and gene expression. Therefore, studies on the effects of salinity on fish have mainly focussed on osmoregulatory organs (gill, intestine and kidney) (Havird et al. 2016; Zhang et al. 2016). Although the liver is not an osmoregulatory organ, it is the major source of energy for osmoregulatory organs. In fish, the liver is important for the metabolism of sugars, fats, proteins, vitamins, hormones, and other components, as well as detoxification, secretion of bile, immune defences, and coagulation, hence the intense focus on this organ in studies on fish metabolism (Cuesta et al. 2005; Singh et al. 2017; Wu et al. 2017). Although energy metabolism in the liver is likely enhanced during salinity adaptation, little is known about the reorganisation of metabolism in this organ during this process in fish. Previous studies on osmoregulation and liver metabolism focused on physiological processes related to osmotic pressure, and energy production and expenditure, but gene expression profiling is not widely reported. Therefore, the present study focused on expression profiling of the liver in grouper exposed to low salinity stress. Transcriptomic analysis can probe gene expression, genetic structures and functions at a genome-wide scale, which is likely to be useful for elucidating and analysing the mechanisms underlying salinity stress adaptation in *E. moara*.

In recent years, transcriptomic analysis of fish in response to salinity stress has mainly focused on genes and enzymes related to osmotic organs and osmotic pressure regulation (Havird et al. 2016), and studies on liver metabolism have not yet been reported. The effects of low salinity stress on gene expression in the liver of *E. moara* were investigated for the first time in this study using the Illumina digital gene expression system. The results showed that the number of DEGs increased with increasing duration of low salinity stress, suggesting this species constantly adjusts in order to adapt to low-salinity environments. Within the first 24 h of low-salinity stress, although the number of DEGs increased continuously, the total number was only 35, 48 and 70 at 2, 6 and 24 h, respectively. By contrast, after low-salinity stress for 48 h, 3141 DEGs were identified, which indicates that sustained low-salinity stress has a significant effect on metabolism in fish. In contrast to the results of this study, Jian et al. (2017) found that the number of DEGs initially increased then decreased with prolonged salinity stress (30, 60 and 90 min). Other studies (Gibbons et al. 2017; Jian et al. 2017) found that whether comparing between salinity levels or between salinity stress duration, only a relatively small number of DEGs were identified, consistent with the results of the present study, in which only 2 DEGs were present in all time point groups.

Real-time PCR is widely used to indicate the reliability and accuracy of RNA-seq data, and while both techniques provide information on gene expression levels, analysis of the functions of DEGs and their associated pathways can be fruitful. GO can be used to define and characterise the functions of DEGs in terms of biological process and cellular components based on the relevant molecular functions of the gene products (Meng et al. 2015; Nie et al. 2016). GO analysis involves annotating unknown genes with known functions from similar genes in other species based on molecular function, biological process and cellular component categories. In the present study, the number of GO terms belonging to the biological process was much larger than those belonging to the molecular function and cellular component categories. With increasing low-salinity stress duration, the number of GO terms in all three categories was increased, and the significance (-log2[*p-value*]) was also increased. Among the terms, those related to plastid and chloroplast cellular component sub-categories were the most abundant. These results suggest the metabolism in the liver of *E. moara* was significantly affected by the duration of low-salinity stress, leading to changes in liver cell components.

In addition to GO analysis, KEGG pathway analysis was performed to elucidate the probable functional status of assembled transcripts. KEGG analysis can provide a more comprehensive and complex analysis of cellular metabolic pathways and processes such as signal transduction, enzymatic reactions, and membrane transport, potentially providing greater biological information (Zhang et al. 2017). KEGG pathway analysis has been widely used to study the effects of salinity stress on fish, but most studies have focused on osmotic pressure regulation (Thanh et al. 2014; Zhang et al. 2016), while liver metabolism has been largely ignored. Our results showed that within the first 24 h of low-salinity stress, KEG pathways were not significantly altered (-log2[*p-value*]). However, after 48 h of low-salinity stress, KEGG pathways were altered significantly, and metabolic pathways were the most different, indicating damage to liver tissue following prolonged exposure to low-salinity conditions.

In conclusion, the results of this study provide a first glimpse into the changes in global gene expression in the liver of *E. moara* that occur in response to low salt stress. A total of 35, 48, 70 and 3141 DEGs were identified after 2, 6, 24 and 48 h of exposure to low-salinity conditions. GO and KEGG enrichment analyses revealed that the expression of genes associated with metabolism were influenced most significantly by low-salinity stress. These pathways and gene expression patterns expand our understanding of the molecular mechanisms underpinning the metabolic changes that occur in response to salinity stress in this important aquaculture species.
